# InPrePPI: an integrated evaluation method based on genomic context for predicting protein-protein interactions in prokaryotic genomes

**DOI:** 10.1186/1471-2105-8-414

**Published:** 2007-10-26

**Authors:** Jingchun Sun, Yan Sun, Guohui Ding, Qi Liu, Chuan Wang, Youyu He, Tieliu Shi, Yixue Li, Zhongming Zhao

**Affiliations:** 1 Virginia Institute for Psychiatric and Behavioral Genetics and Department of Psychiatry, Virginia Commonwealth University, Richmond, VA 23298, USA; 2 Bioinformation Center, Shanghai Institutes for Biological Sciences, Chinese Academy of Sciences, Shanghai 200031, China; 3 Graduate School, Chinese Academy of Sciences, Shanghai 200031, China; 4 School of Life Sciences and Technology, Shanghai Jiaotong University, Shanghai 200240, China; 5 Department of Human Genetics, Virginia Commonwealth University, Richmond, VA 23298, USA; 6 Center for the Study of Biological Complexity, Virginia Commonwealth University, Richmond, VA 23284, USA

## Abstract

**Background:**

Although many genomic features have been used in the prediction of protein-protein interactions (PPIs), frequently only one is used in a computational method. After realizing the limited power in the prediction using only one genomic feature, investigators are now moving toward integration. So far, there have been few integration studies for PPI prediction; one failed to yield appreciable improvement of prediction and the others did not conduct performance comparison. It remains unclear whether an integration of multiple genomic features can improve the PPI prediction and, if it can, how to integrate these features.

**Results:**

In this study, we first performed a systematic evaluation on the PPI prediction in *Escherichia coli *(*E. coli*) by four genomic context based methods: the phylogenetic profile method, the gene cluster method, the gene fusion method, and the gene neighbor method. The number of predicted PPIs and the average degree in the predicted PPI networks varied greatly among the four methods. Further, no method outperformed the others when we tested using three well-defined positive datasets from the KEGG, EcoCyc, and DIP databases. Based on these comparisons, we developed a novel integrated method, named InPrePPI. InPrePPI first normalizes the *AC *value (an integrated value of the accuracy and coverage) of each method using three positive datasets, then calculates a weight for each method, and finally uses the weight to calculate an integrated score for each protein pair predicted by the four genomic context based methods. We demonstrate that InPrePPI outperforms each of the four individual methods and, in general, the other two existing integrated methods: the joint observation method and the integrated prediction method in STRING. These four methods and InPrePPI are implemented in a user-friendly web interface.

**Conclusion:**

This study evaluated the PPI prediction by four genomic context based methods, and presents an integrated evaluation method that shows better performance in *E. coli*.

## Background

Uncovering all protein-protein interactions (PPIs), or, the interactome, of an organism is essential for understanding its complex biological processes [1,2]. Recently, many high-throughput experimental and computational methods have been developed and applied to model organisms such as *Escherichia coli *(*E. coli*), yeast, and humans [3-10]. High-throughput experimental methods can directly detect the set of PPIs in a genome, but the capacity to identify PPIs is still limited by present technology. Computational approaches, which usually mine and then utilize the features from the known PPIs and the genomic information from one or multiple genomes, can largely meet this strong demand [11]. The major limitation in both the computational and experimental approaches is their uncertain confidence in the identification of PPIs, with high false-positive and false-negative rates [12,13].

Genomic context information has been frequently used in the computational methods for PPI prediction. There are four major genomic context based methods: the phylogenetic profile method [14], the gene cluster method [3], the gene fusion method [15], and the gene neighbor method [16]. Each method mainly utilizes one specific genomic context feature; thus, its prediction has biases towards the information it relies on [12]. There is one comparison of the phylogenetic profile, gene fusion, and gene neighbor methods, suggesting that the gene neighbor method might outperform the other two [17,18]. To date, there have been no other systematic evaluations of these four methods. It is likely that an integration of these methods would take advantage of different genomic features and thus outperform each of these four methods [12]. Indeed, investigators now realize the importance of integration [19,20]. The integration strategy has been applied in two methods: the joint observation method [3,14,21] and STRING [22]. The joint observation method selects the PPIs that are predicted or identified by more than one method [10,21]. Its rationale is based on the understanding that the confidence of PPI prediction relies on the amount of supporting evidence, and that the confidence increases with more evidence (i.e., methods). This strategy was successfully demonstrated in Uetz *et al*. [23] and von Mering *et al*. [12]. However, the joint observation method results in a strong decrease of the coverage, especially when the number of methods becomes large. Since an efficient approach to inferring PPIs needs to consider both coverage and accuracy, the joint observation method has limited applications [12,24]. STRING calculates a combined score for each pair of proteins assuming that the features from various sources are independent [22]. While this scoring algorithm has been implemented in the STRING database, there is no evaluation on the improvement of PPI prediction.

In this study, we first performed a systematic evaluation on the prediction efficacy of these four genomic context based methods by using three gold standards of positive datasets obtained from the KEGG [25], EcoCyc [26], and DIP databases [27], respectively. We used *E. coli *K12 in this study because it is the most studied prokaryotic organism and its protein annotations are available in several databases. Our evaluation indicated that there is no consensus among these methods and no method could outperform the others in all tests. Based on these comparisons, we developed a new method to integrate the features used in all four methods. We named the method InPrePPI (an Integrated method for Prediction of Protein-Protein Interactions). InPrePPI first calculates a score for each protein-protein pair predicted by each method, then optimally weighs the score, and finally obtains an integrated score. Based on the integrated score, InPrePPI extracts the PPIs with high confidence from all of the predicted protein pairs. Our comparison of InPrePPI with the joint observation method and STRING indicates that InPrePPI in general outperforms the others. Finally, we implemented the four genomic context based methods and InPrePPI in a user-friendly platform-independent system.

## Results

### Comparison of the PPIs predicted by the four methods

We performed a systematic evaluation on the prediction of PPIs in *E. coli *K12 by four genomic context based methods: the phylogenetic profile, gene cluster, gene fusion and gene neighbor methods. Throughout the rest of this paper, we will abbreviate these four methods as "PPM", "GCM", "GFM", and "GNM", respectively. The prediction results are summarized in Table 1. The number of predicted PPIs was 45,437 (PPM), 2,437 (GCM), 6,728 (GFM), and 3,595 (GNM), respectively. These numbers varied greatly; for example, the number of PPIs predicted by the PPM is approximately 19 times more than was predicted by the GCM.

**Table 1:** Protein-protein interactions predicted by four methods

Method	Number of PPIs	Number of proteins involved	Average degree	Number of PPIs covered by two methods			
				
				PPM	GCM	GFM	GNM
PPM	45,437	2,124	21.4				
GCM	2,437	2,102	1.2	449			
GFM	6,728	1,254	5.4	1,532	134		
GNM	3,595	3,901	0.9	300	1,155	124	
Total^a^	54,911	4,040	13.6				

^a^Number of non-redundant PPIs predicted by the four methods.

We next examined the average degree for the PPIs predicted by the four methods. The degree is the most elementary characteristic of a node in a biological network [28]. If the average degree in the predicted network is much lower than the expected, it may reflect that the prediction does not have a good coverage of the PPIs in the genome. Conversely, if it is much higher than the expected, it may reflect many false positive results in the prediction (i.e., low accuracy). Note that this comparison does not directly test the performance. We measured the average degree by the average number of links in the predicted PPIs. The average degree was close to 1 in the GCM or GNM, remarkably lower than that in the PPM (21.4) or GFM (5.4) (Table 1). According to the previous estimations, an average degree should be in a range of 2 to 10 links for each protein in a typical functioning cell [29,30]. Thus, it seems that only the GFM had a reasonable average degree. Overall, the prediction of PPIs varied greatly among these four genomic context methods.

Finally, we examined the PPIs that were similarly predicted by more than one method. A total of 1,155 PPIs were predicted by both the GCM and GNM. They accounted for 47% of the total predicted PPIs by the GCM and 32% by the GNM (Table 1). For the PPIs predicted by the GFM and PPM, 1,532 overlapped, which accounted for 23% of the total PPIs by the GFM and 3% by the PPM, respectively. The number of overlapped PPIs in the remaining comparisons between two methods was smaller (Table 1). Furthermore, there were only 298 PPIs that were predicted by three or more methods. Of those 298 PPIs, 55 were predicted by all four methods. The comparison suggests that (1) GCM and GNM, which likely share some common genetic context information, have similar predictions of PPIs to some extent, and (2) there was no consensus in the prediction of PPIs by these methods that utilize different features of genomic context. The lack of consensus in prediction by different methods was similarly reported in the previous study [17], implying that they could complement each other.

### Biological biases of the PPIs predicted by the four methods

We further compared the features of these four methods by evaluating the performance of PPI prediction using three well-defined datasets from the KEGG, EcoCyc, and DIP databases. The KEGG dataset included pathway information, the EcoCyc included protein complexes, and the DIP included the protein interactions with evidence. The performance of each method was measured by an *AC *value, which is an integrated value of the accuracy and coverage (see Methods), because an assessment of the prediction needs to consider both accuracy and coverage [12].

**Figure 1:**
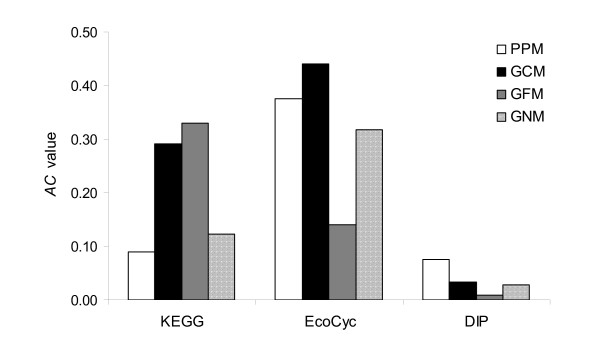
**Comparison of PPI prediction by the four methods using the KEGG, EcoCyc, and DIP datasets**. Performance of the prediction was measured by *AC *value.

Figure 1 shows the *AC *values of the four methods using all three datasets. The results can be summarized in the following three points. First, among the four methods, the GFM had the highest *AC *value in the KEGG dataset; in contrast, it had the lowest value in the EcoCyc and DIP datasets. Further examination of the KEGG dataset, which included 1,386 *E. coli *proteins, found a total of 117 pathways, of which 103 were in the category of metabolism. This indicates that most proteins in the KEGG dataset are involved in metabolism. The preference of the GFM in metabolic proteins is consistent with Tsoka and Ouzounis' previous report [31]; thus, it suggests that the GFM performs well in the prediction of PPIs involved in metabolisms. Second, the GCM had the highest *AC *value in the EcoCyc dataset, which is consistent with the concept that genes in the same operon often encode proteins involved in the protein complexes. Third, in contrast to the GFM and GCM, the PPM had the highest *AC *value in the DIP dataset but the lowest value in the KEGG dataset. This suggests that the PPM may be suitable for prediction of PPIs involved in protein interactions but not in the pathways. Overall, no method outperformed the others among these three datasets.

**Figure 2:**
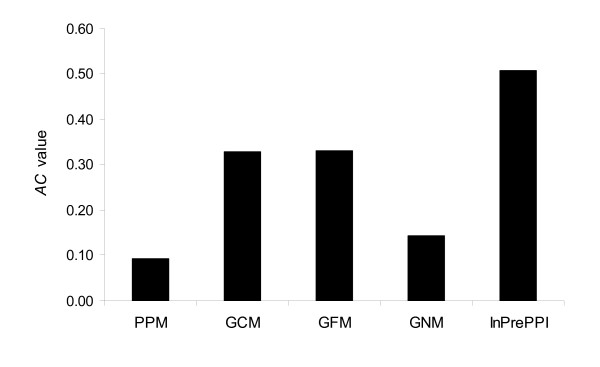
**Comparison of PPI prediction by four individual methods and InPrePPI**. The combined protein pairs in the KEGG, EcoCyc, and DIP datasets were used in the four methods and InPrePPI_high dataset was used in InPrePPI.

We combined all non-redundant protein pairs in the KEGG, EcoCyc, and DIP datasets and calculated the *AC *values for these methods. The *AC *values in the GCM and GFM were similar and higher than those in the PPM and GNM (Figure 2).

### InPrePPI method

**Figure 3:**
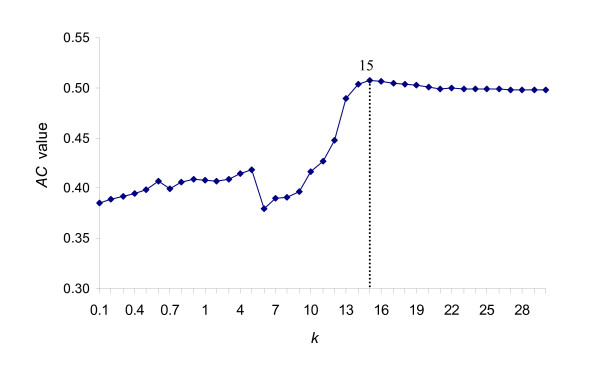
PPI prediction by InPrePPI with different *k *values.

The results in the above two sections indicate that each method has its own superiority and no one outperforms the others. Thus, we developed a new method, InPrePPI, which weighs the genomic context information utilized in these four methods and integrates it into a system that can optimize the prediction. Specifically, the InPrePPI uses the *AC *values of the four methods based on three positive datasets (KEGG, EcoCyc, and DIP). A constant, *k*, is used in the integration process (see Methods). This *k *can be obtained by a heuristic approach. We tested *k *values from 0 to 1 (in an interval 0.1) and from 1 to 30 (in an interval 1). For each *k*, we calculated the integrated score ($\hat{S}$) for each protein pair and then obtained a set of PPIs with the highest scores (InPrePPI_high, see Methods). The optimal *k *value is found when it results in the highest *AC *value in the InPrePPI_high class. Figure 3 shows the *AC *values using different *k *values and the InPrePPI_high class. The *AC *values increased when *k *increased until *k *reached 15. Thus, the optimal *k *was set to 15.

When *k *= 15, we assigned an integrated score to each of the 54,911 pairs predicted by the four methods (Table 1). These 54,911 pairs were separated into three classes based on the prediction confidence: InPrePPI_high (1,194 pairs), InPrePPI_medium (5,403), and InPrePPI_low (48,314). The data are available at InPrePPI web site [32] or upon request.

### Comparison of InPrePPI with other methods

We first compared the PPI prediction by InPrePPI with the four individual methods. The *AC *value was higher in InPrePPI than each of the four methods (Figure 2).

Next, we compared the performance of InPrePPI with the two existing integrated methods: the joint observation method (JOM) [21] and STRING [22]. In JOM, we calculated the accuracy and coverage for the PPIs that were predicted by at least one, two, three, or four methods (PPM, GCM, GFM, and GNM), respectively, using three positive datasets (KEGG, EcoCyc, and DIP). Confidence of the PPI prediction is expected to increase when a pair is simultaneously predicted by multiple methods. This was confirmed, i.e., the accuracy increased from 8.79% by at least one method (JOM_≥1_) to 78.18% by all the four methods (JOM_4_) using the KEGG dataset (Table 2). However, the coverage values decreased drastically. In the KEGG dataset, the coverage value decreased from 10.98% (JOM_≥1_) to only 0.1% (JOM_4_). A similar pattern was observed in the EcoCyc and DIP datasets (Table 2). In InPrePPI, when the confidence level of the three classes (InPrePPI_high, InPrePPI_medium, and InPrePPI_low) increased, the accuracy also increased in all three positive datasets, whereas the coverage decreased in the KEGG and DIP datasets. However, the extent of the decrease was much weaker than that in the JOM. Interestingly, the coverage of InPrePPI increased greatly in the EcoCyc dataset. We noted that the accuracy values in the InPrePPI_high class were lower than those in JOM_4 _and JOM_≥3_, but higher than those in JOM_≥1 _and JOM_≥2_. Because numbers of PPIs in the JOM_4 _and JOM_≥3 _were small, its applications are limited. Overall, InPrePPI outperforms JOM.

**Table 2:** Accuracy and coverage in three integrated methods

		KEGG		EcoCyc		DIP	
		
	Number of PPIs	Accuracy (%)	Coverage (%)	Accuracy (%)	Coverage (%)	Accuracy (%)	Coverage (%)
Joint observation method (JOM)							
JOM_4_^a^	55	78.18	0.10	32.73	2.65	25.45	0.44
JOM_≥3_	298	60.74	0.41	32.89	14.45	12.42	1.17
JOM_≥2_	2,933	38.70	2.58	9.00	38.94	2.35	2.18
JOM_≥1_	54,911	8.79	10.98	0.85	69.17	0.49	8.58
							
STRING							
High^b^	2,279	24.62	1.28	13.43	42.33	3.20	2.31
Medium	4,458	5.74	0.58	1.39	7.08	0.31	0.44
Low	9,970	2.18	0.49	0.17	2.21	0.11	0.35
							
InPrePPI							
High^c^	1,194	45.73	1.24	18.84	33.19	4.69	1.77
Medium	5,403	27.93	3.43	2.24	17.85	0.91	1.55
Low	48,314	5.73	6.30	0.25	18.14	0.34	5.25

^a^The predicted PPIs covered by at least one (JOM_≥1_), two (JOM_≥2_), three (JOM_≥3_) or four (JOM_4_) methods.

^b^The predicted PPIs in the high, medium and low confidence in STRING [22].

^c^The predicted PPIs in the high, medium and low confidence in InPrePPI (see Methods).

**Figure 4:**
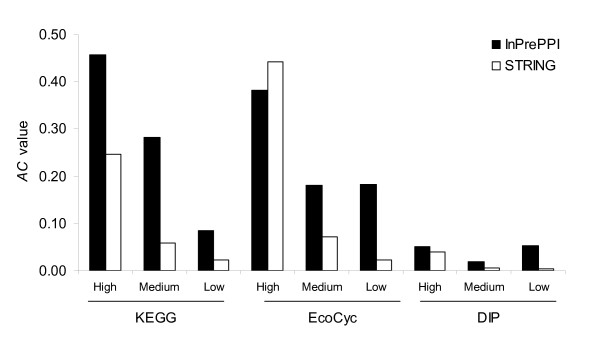
**Comparison of PPI prediction by InPrePPI and STRING using the KEGG, EcoCyc, and DIP datasets**. The data were separated into three groups with the high, medium, and low confidence.

The PPI data predicted by the methods in STRING were retrieved from the STRING database (see Methods) and used in our comparison. These data were separated by the STRING algorithm into three groups based on the confidence level (high, medium, or low) [22]. Table 2 shows that InPrePPI had consistently higher accuracy values than STRING. The coverage values in InPrePPI were higher than or close to those in STRING, except for two subcategories (InPrePPI_high class in EcoCyc and DIP). We further compared the *AC *values in three classes. Excluding the high confidence class in the EcoCyc dataset, all *AC *values in InPrePPI were higher than those in STRING (Figure 4). In fact, in the high confidence class of the EcoCyc dataset, InPrePPI had a slightly smaller *AC *value than STRING (Figure 4). This comparison indicates that InPrePPI overall performed better than the prediction in STRING.

**Figure 5:**
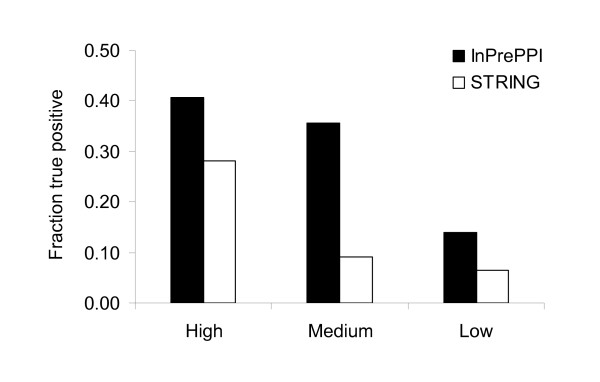
**Comparison of PPI prediction by InPrePPI and STRING using the COG annotation data**. A predicted pair is treated as a true positive when its two proteins are within the same COG well-characterized category.

Protein annotations of Clusters of Orthologous Groups (COG) have been used in the assessment of PPI prediction [33,34]. Here we used COG annotations for *E. coli *K12 proteins to assess the prediction performance by InPrePPI and STRING. There are 25 COG functional categories, including 22 well-characterized and 3 poorly characterized or unknown categories. A predicted pair is counted as a true positive when its two proteins are within the same COG well-characterized category and as a false positive otherwise. The fractions of true positives were 0.408 (487 true positives over the 1,194 predicted pairs, 487/1,194) for InPrePPI_high, 0.356 (1,926/5,403) for InPrePPI_medium, and 0.139 (6,722/48,314) for InPrePPI_low, respectively, while the corresponding fractions in STRING were 0.280 (639/2,279) for STRING_high, 0.091 (407/4,458) for STRING_medium, and 0.065 (644/9,970) for STRING_low. Based on this metric, InPrePPI had better prediction performance than STRING (Figure 5).

### Implementation

A web-based, user-friendly application (InPrePPI) for PPI prediction was implemented by Java. This InPrePPI web interface [32] allows the user to predict PPIs using one of the four methods (PPM, GCM, GFM, and GNM) or InPrePPI. If the user chooses InPrePPI, the application first predicts PPIs using the four methods and then assigns an integrated score ($\hat{S}$) to each pair of the predicted PPIs. The user has the option to set or modify parameters such as BLASTP E-value, target organism, or list of reference organisms. This package can be downloaded at no cost from the web site and installed in a local computer. Because the system was designed to provide flexibility in PPI prediction, the data are not pre-computed. This may lead to a long computation time; therefore, we recommend that the user retrieve the results via email or run it directly in a local computer.

## Discussion

Many biological features have been explored in the prediction of protein-protein interactions and it has been found that there is limited prediction power when utilizing only one genomic feature. Investigators are now moving toward integration [12,22,35]. A systematic assessment of the existing methods is a prerequisite to an effective integration. In this study, we focused on four major methods (PPM, GCM, GFM, and GNM) that utilize genomic context information. Each method characterizes in its own way. We hypothesized that an efficient integration of these four major methods would improve prediction performance. We first performed extensive comparisons of these four methods using three positive datasets (KEGG, EcoCyc, and DIP). We found that these four methods lacked consensus but complemented each other to some extent. Based on these comparisons, we developed an integrated method, InPrePPI, which optimally weighs the scores of protein pairs predicted by the four methods. Our performance comparison indicates that InPrePPI outperforms each individual method (Figure 2) and, in general, the other two integrated methods: the JOM and STRING (Table 2, Figures 4 and 5).

However, InPrePPI did not outperform the JOM or STRING in all tests. In the JOM, the accuracy values were higher for the PPIs that were consistently predicted by at least three methods. Such high values were reached by dramatically decreasing the coverage. This makes JOM impractical when multiple methods or supporting evidence is employed. InPrePPI does not have this limitation because it uses an integration score, rather than an intersection of multiple data. Compared to STRING, InPrePPI had consistently higher accuracy values and its coverage values were higher or close, in most cases, except in the high confidence class of the EcoCyc and DIP datasets. In the latter two cases, the difference was not as remarkable as it was in the comparison between the JOM and InPrePPI. For example, the coverage value in InPrePPI was 33.19% in the high confidence class of EcoCyc; this is comparable to the 42.33% in STRING but much higher than the 2.65% in the JOM_4 _(Table 2). When we considered both the accuracy and coverage values, InPrePPI outperformed STRING in all tests except in the high confidence class of EcoCyc (Figure 4). Furthermore, our independent test using COG annotations indicates that the fractions of true positives in InPrePPI were consistently higher than those in STRING in all three classes of predicted PPIs (Figure 5).

The STRING database provides a comprehensive, high quality collection of protein-protein associations for a large number of organisms [22]. The association data were compiled from high-throughput experimental data, mining of other databases and literature, and the predicted PPIs by genomic context approaches. We demonstrated that InPrePPI has an overall better performance than the prediction methods (phylogenetic co-occurrence, conserved neighborhood, and gene fusion methods) in STRING. However, InPrePPI is limited to the evaluation and prediction of protein-protein pairs based on the genomic context features and its web site provides only prediction function rather than a comprehensive evidence collection. While the STRING database provides a powerful system for proteomics research, the amount of PPI data collected by the high-throughput experiments, or from the existing literature, is still very limited at present in most organisms in nature and is likely to be limited for some time. Computational approaches are expected to play an important role in uncovering the interactomes of most genomes. Although one recent study failed to improve the prediction by adding more features [35], the InPrePPI method demonstrates that an integration, if appropriate, can improve prediction power. Thus, our integrated method based on the genomic context, which is to be further optimized and enhanced, can be applied to the prediction of PPIs in many other (prokaryotic) genomes and also integrated into the comprehensive database such as STRING.

InPrePPI integrates four genomic context based methods. These four methods are currently the best computational methods for prokaryotic genomes. This implies that InPrePPI may be applied to the discovery of PPIs at least in prokaryotic genomes. InPrePPI uses a constant, *k*, to normalize the *AC *value and calculate the weight of each method. This constant depends on the data used and the methods integrated and can be obtained by a heuristic approach. When true positives are available in a genome, the optimal *k *value and weight of each method can be directly obtained by the method in this study. To predict PPIs in a genome without true positive data, which is very challenging at present and always relies on the knowledge in other well-studied organisms, we may use the optimal *k *value and the weight available in *E. coli *or any other genome that is related to the target genome and then refine it after some of the predicted PPIs have been validated (i.e., true positives). InPrePPI may be extended to eukaryotic genomes as well. Recent assessments of phylogenetic profiling in the *E. coli *and yeast confirmed the similar strategy of reference organism selection in the construction of phylogenetic profiles [36-38] and indicate that phyletic patterns of proteins in prokaryotes alone are adequate to predict functional linkages between proteins in prokaryotic and eukaryotic genomes [37]. Some studies have reported that neighboring genes have similar expression patterns in higher eukaryotes, implying possible interactions [39-41]. Qi *et al. *[13] found that gene co-expression is consistently the most important feature in their comprehensive evaluation of PPI prediction in yeast using an integrated framework, which supports the previous finding that the most obvious co-expression comes from permanent complexes such as ribosome and proteasome [42,43]. Therefore, we may consider both the genomic context information and the gene co-expression data when we extend InPrePPI to eukaryotic genomes.

We used the gold standards of positives to evaluate the PPI prediction methods. In previous studies, positive data was selected from the standardized SWISS-PROT keywords [3,30], the metabolic map in KEGG [22], the pathway information in COG [33], or the protein complexes [12]. So far, there has been no complete biological database to serve as a gold standard of positives. To avoid a biased selection of positive data, we used three well-documented datasets: (1) biological pathway information from KEGG, (2) protein complexes from EcoCyc, and (3) protein-protein interactions identified by experiments from DIP. The prediction performance of each method varied among these three datasets (Figure 1), suggesting that the selection of positive control data should be made carefully and should consider the types of interactions.

## Conclusion

Computational prediction will play a major role in the exploration of the interactomes of many genomes. However, a computational method that relies on one specific genomic context feature has limited power in PPI prediction. We believe that an integration approach, which efficiently takes advantage of the different genomic features, will outperform individual methods. In this study, we first evaluated the prediction performance of the four major genomic context based methods (PPM, GCM, GFM, and GNM), then we developed a novel integrated method (InPrePPI) based on the comparisons of these four methods in three datasets (KEGG, EcoCyc, and DIP). We demonstrated that InPrePPI, which is an evaluation rather than prediction method, outperforms these four individual methods and, in general, the other two existing integrated methods (JOM and STRING).

## Methods

### Data sources

We downloaded genes and their annotations (e.g., name, length, orientation, and protein sequence) in the 226 available complete genomes from the NCBI RefSeq database [44]. We chose *E. coli *K12 as the target organism and the remaining 225 organisms as reference organisms. The predicted operons in prokaryotes were downloaded from SHOPS [45]. We downloaded the PPI data in STRING from its web site [46] and then retrieved those PPIs predicted by the methods (phylogenetic co-occurrence, conserved neighborhood, and gene fusion) in STRING. We retrieved the COG annotations for *E. coli *K12 proteins from the NCBI *E. coli *K12 genome database [47].

### Four genomic context based methods

We predicted PPIs using the genome datasets collected above by four genomic context based methods: the phylogenetic profile method [14], the gene cluster method [3,33], the gene fusion method [15], and the gene neighbor method [16]. We briefly describe these methods below; the details of these methods are provided in their original publications.

In the phylogenetic profile method, we used the refined method described in Sun *et al*. [48] to obtain an optimal reference organism set from the 225 available complete genomes. The homology of a protein was identified by the BLASTP program [49] with an E-value < 1 × 10^-4^. We chose the E-value threshold of 1 × 10^-4 ^because of its optimal performance in our previous evaluation [48]. The phylogenetic profile for each *E. coli *protein was then constructed and assessed using the mutual information (MI) value calculated by the method in Date and Marcotte [50]. The MI value of each protein pair reflects the confidence level of the link between the two proteins. To identify the candidate interactions, we calculated the threshold of mutual information (TMI) values using the method in Sun *et al*. [48]. A pair of proteins was considered to interact when its MI value was higher than the TMI value.

In the gene cluster method, the genes that belong to one operon in *E. coli *and have homologues also belonging to another operon in the reference genome(s) were considered to have functional links with each other. In the gene fusion method, two or more proteins were identified to be functionally linked when they were not encoded by neighboring genes in *E. coli *but were uniquely homologous to a single protein in a reference organism [15]. In the gene neighbor method, we identified those genes that were located as neighbors (i.e., physically linked) among multiple genomes [51].

Identification of each protein pair is based on the genomic context within a variety of genomes; some were closely related while the others were not. Thus, we assigned a score to each protein pair by the evolutionary distance between the target organism and the reference organism where the pair was present. We used the conserved 16S rRNA gene to estimate the evolutionary distance between *E. coli *and the other prokaryotic genomes. We downloaded the 16S rRNA gene sequences in *E. coli *and the other 211 prokaryotic genomes from NCBI [44]. We then aligned them using the ClustalW program [52]. After a manual check and adjustment of the alignments, we estimated the genetic distance using the PHYLIP package [53]. Finally, we calculated the score for each protein pair, which is the sum of the evolutionary distances between *E. coli *and the other genomes where the protein pair was present.

### Gold standard positives and negatives

Assessment of the prediction performance in a computational method needs control datasets including gold standard positives (i.e., proteins that do interact) and gold standard negatives (i.e., proteins that do not interact). We collected three datasets for gold standard positives from the following established databases: (1) pathway information from the KEGG database [25], (2) protein complexes from the EcoCyc database [26], and (3) protein-protein interactions from the DIP database (version: Ecoli20060116) [27]. In the EcoCyc database, we downloaded the file 'protcplxs.col'; this file lists the genes that encode the subunits of the complex. Among these databases, the proteins that were involved in the same complex or pathway were compiled and served as the positives. We used the data in KEGG Orthology (KO) [54] for gold standard negatives. We first removed all of the proteins that were involved in more than one functional category at the first level of KO. Then, we selected two proteins each time from the remaining proteins to form a pair. Because the two proteins in each pair were from different functional categories at the first level, they served as negative controls, assuming that two proteins from different broad functional categories did not interact [12]. Table 3 summarizes the processed positive and negative functional association data used in this study. No overlap was found between the negative and positive data.

**Table 3:** Summary of the positive and negative control data

Category	Number of protein pairs	Overlap			Source
			
		KEGG	EcoCyc	DIP	
KEGG	43,937				KEGG [25]
EcoCyc	678	506			EcoCyc (8.0) [26]
DIP	3,159	141	54		DIP (Ecoli20060116) [27]
Positives^a^	47,105				KEGG + EcoCyc + DIP
Negatives	376,874				KO [54]

^a^The non-redundant pairs in the KEGG, EcoCyc, and DIP datasets. There is no overlap between negatives and positives.

### Evaluation of PPI prediction

To assess the performance of PPI prediction, we calculated the accuracy and coverage in each method and then obtained an integrated value (*AC *value) by the following equations:

$$Accuracy=\frac{TP}{TP+FP},$$

$$Coverage=\frac{TP}{TP+FN},$$

$$AC=\sqrt{{\left(Accuracy\right)}^{2}+{\left(Coverage\right)}^{2}}.$$

In the equations above, TP (true positive) is the number of the predicted PPIs that were found in the positive control dataset, FP (false positive) is the number of the predicted PPIs that were not found in the positive control dataset, and FN (false negative) is the number of PPIs in the positive control dataset that failed to be predicted by the method.

### InPrePPI

InPrePPI weighs and integrates the scores of each protein pair obtained by the four methods: PPM, GCM, GFM, and GNM. There are three steps to calculate an integrated score for each protein pair. First, the *AC *value for each method is normalized by

$$\begin{array}{cc} AC{'}_{i,j}=e^{\left(-k/AC_{i,j}\right)} & AC'\in [0,1] \end{array}$$

where *k *is a positive constant whose optimal value can be empirically obtained by comparing the *AC *values using the predicted PPIs with high confidence (InPrePPI_high, see below and the Results), *i *is an index of positive datasets (i.e., KEGG, EcoCyc, and DIP), and *j *is an index of methods (i.e., PPM, GCM, GFM, and GNM). Second, for each method *j*, we calculate the weight (*W*_*j*_) by

$$\begin{array}{cc} W_{j}=1-\prod_{i=1}^{3}\left(1-AC{'}_{i,j}\right) & W_{j}\in [0,1]. \end{array}$$

Third, for each pair of proteins, an integrated score ($\hat{S}$) is calculated by

$$\begin{array}{cc} \hat{S}=1-\prod_{j=1}^{4}\left(1-W_{j}\times S_{j}\right) & \hat{S}\in [0,1] \end{array}$$

where *S*_*j *_is the score of the pair by method *j*.

We categorized the predicted PPIs into three groups according to their prediction confidence. We first obtained two average scores to serve as the cutoff values: Score_P, the average score among the predicted protein pairs whose interactions are known to be true (i.e., in the positive dataset), and Score_N, the average score among the predicted protein pairs whose interactions are known to be false (i.e., in the negative dataset). The predicted protein pairs whose scores were higher than Score_P were considered to have high confidence and were categorized into the InPrePPI_high class. The predicted protein pairs whose scores were lower than Score_N were considered to have low confidence and were categorized into the InPrePPI_low class. The remaining protein pairs, whose scores were between Score_N and Score_P, were categorized into the InPrePPI_medium class.

## List of abbreviations

PPI: protein-protein interaction

InPrePPI: an integration method for prediction of protein-protein interactions

PPM: phylogenetic profile method

GCM: gene cluster method

GFM: gene fusion method

GNM: gene neighbor method

JOM: joint observation method

## Authors' contributions

JS participated in the method development, prepared the data, carried out the data analysis, and contributed to the writing of the manuscript. YS developed the InPrePPI web system. GD contributed to the web system development and data analysis. QL, CW, YH, and TS participated in its design and coordination. YL conceived of the study and participated in the method development. ZZ participated in the method development and data analysis and contributed to the writing of the manuscript. All authors read and approved the final manuscript.
